# Hepatitis B prevalence and incidence in the fishing communities of Lake Victoria, Uganda: a retrospective cohort study

**DOI:** 10.1186/s12889-021-10428-1

**Published:** 2021-02-23

**Authors:** Paul Kato Kitandwe, Enoch Muyanja, Teddy Nakaweesa, Annet Nanvubya, Ali Ssetaala, Juliet Mpendo, Brenda Okech, Bernard S. Bagaya, Noah Kiwanuka, Matt A. Price

**Affiliations:** 1 MRC/UVRI & LSHTM Uganda Research Unit, P.O. Box, 49, Entebbe, Uganda; 2 UVRI-IAVI HIV Vaccine Program, Entebbe, Uganda; 3 Department of Immunology and Molecular Biology, School of Biomedical Sciences, College of Health Sciences Makerere University, Kampala, Uganda; 4 Department of Epidemiology and Biostatistics, School of Public Health, College of Health Sciences Makerere University, Kampala, Uganda; 5 International AIDS Vaccine Initiative, New York, NY USA; 6 Department of Epidemiology and Biostatistics, University of California San Francisco, San Francisco, CA USA

**Keywords:** Hepatitis B prevalence, Hepatitis B incidence, HBV prevalence, HBV incidence, Lake Victoria fishing communities

## Abstract

**Introduction:**

Hepatitis B is a serious potentially fatal hepatocellular disease caused by the hepatitis B virus. In the fishing communities of Lake Victoria Uganda, the hepatitis B virus infection burden is largely unknown. This study assessed the prevalence and incidence of hepatitis B in these communities.

**Methods:**

This was a retrospective cohort study that tested serum samples collected from 13 to 49-year-old study participants that were residing in two Ugandan Lake Victoria fishing communities of Kasenyi (a mainland) and Jaana (an island). The samples were collected between 2013 and 2015 during the conduct of an HIV epidemiological cohort study in these communities. A total of 467 twelve-month follow-up and 50 baseline visit samples of participants lost to follow-up were tested for hepatitis B serological markers to determine prevalence. To determine hepatitis B virus incidence, samples that were hepatitis B positive at the follow-up visit had their baseline samples tested to identify hepatitis B negative samples whose corresponding follow-up samples were thus incident cases.

**Results:**

The baseline mean age of the 517 study participants was 31.1 (SD ± 8.4) years, 278 (53.8%) of whom were females. A total of 36 (7%) study participants had hepatitis B virus infection, 22 (61.1%) of whom were male. Jaana had a higher hepatitis B virus prevalence compared to Kasenyi (10.2% vs 4.0%). In total, 210 (40.6%) study participants had evidence of prior hepatitis B virus infection while 48.6% had never been infected or vaccinated against this disease. A total of 20 (3.9%) participants had results suggestive of prior hepatitis B vaccination. Hepatitis B incidence was 10.5 cases/100PY (95% CI: 7.09–15.53). Being above 25 years of age and staying in Jaana were significant risk factors for hepatitis B virus acquisition (AOR 1.6, 95% CI: 1.1–2.2; *p* < 0.01 and 1.4, 95% CI: 1.1–1.8; p < 0.01 respectively).

**Conclusion:**

Hepatitis B virus incidence in Lake Victoria fishing communities of Uganda is very high, particularly in the islands. Interventions to lower hepatitis B virus transmission in these communities are urgently needed.

## Introduction

Hepatitis B is a viral infection caused by the hepatitis B virus (HBV) which leads to acute and chronic liver disease. Chronic liver disease can lead to cirrhosis and associated life-threatening complications such as hepatocellular carcinoma [1]. The World Health Organization (WHO) reported that in 2015, an estimated 887,000 people died from HBV infection-related complications worldwide [2].

Transmission of HBV occurs primarily through percutaneous or mucosal exposure to infected blood or other body fluids [3]. In highly endemic areas, HBV is mainly spread at birth from mother to child or during the first 5 years of life from an infected to an uninfected child [4]. It is estimated that up to 90% of perinatal HBV infections, 20 to 60% of infections occurring between the ages of 1 to 5 years, and 5 to 10% of infections occurring above 5 years become chronic [5]. In low-endemicity areas, most HBV infections occur in adolescents and young adults primarily through sexual or percutaneous exposure [6].

The global prevalence of HBV is estimated at 3.5–3.9% but with wide regional variations [2, 7, 8]. Sub-Saharan Africa has been reported to have the highest HBV endemicity with more than 8% of the adult population estimated to be chronically infected [8]. According to the Uganda Population-based HIV Impact Assessment (UPHIA) 2016–2017 national serosurvey, HBV infection prevalence among Ugandan adults was 4.3% with the northern region of the country having the highest prevalence (4.6%) and the southwest the lowest (0.8%) [9]. In 2015, an estimated 52% of Ugandan adults had evidence of previous or ongoing HBV infection [10].

Adults in the Lake Victoria fishing communities were reported to engage in high-risk sexual behaviors which put them at an increased risk of acquiring sexually transmitted infections [11]. Indeed, high HIV prevalence and incidence rates of 22 to 28% and 3.4–4.9/100 person-years at risk respectively have been reported in these communities [11–14]. Since HIV and HBV share routes of transmission, a high proportion of adults at risk for HIV infection in the Lake Victoria fishing communities might also be at risk of acquiring HBV [15, 16]. Whereas several studies have reported on the prevalence of HBV infection in different Ugandan populations, we are not aware of any that have specifically targeted the Lake Victoria fishing communities. This study therefore sought to investigate the prevalence, incidence, and risk factors of HBV infection among the Lake Victoria fishing communities of Uganda.

## Methods

### Study design and population

This was a retrospective study that used archived serum samples collected during an HIV epidemiological cohort study. The cohort study was conducted in 10 fishing communities of Lake Victoria, Uganda between 2013 and 2015. The study communities were purposively selected based on lack of participation in previous HIV epidemiological studies, geographical representation, and their large size. Our previously conducted research suggested that limited or no risk screening was necessary as HIV risk in fishing communities was widespread [11–13]; Communities were selected based on size and no HIV risk criteria were used to select study participants. The study recruited 13 to 49-year-old males and females randomly selected from an updated census database conducted for all households in these communities before the start of the cohort study. Participants were residents in these communities for at least 6 months at the time the cohort study was conducted. Apart from age and residency eligibility, no other inclusion or exclusion criteria were used to select study participants. For this retrospective HBV study, samples from two of the communities that had the highest study participant retention rate after 12 months of follow-up were used to allow for more power to calculate incidence and to reduce selection bias that could be caused by volunteer loss to follow up. These two communities selected were Kasenyi located on the mainland in Wakiso district and Jaana an island in Kalangala district which had 12-month retention of 93.9 and 93.4% respectively. To further minimize subject selection bias, all volunteer samples collected from the two communities during the conduct of the parent HIV epidemiological cohort study were used.

### Hepatitis B testing and determination of HBV status

Each serum sample was tested on three different HBV tests namely, HBV surface antigen (HBsAg), HBV core antibodies (anti-HBc), and HBV surface antibodies (anti-HBs). Testing was done from the MRC/UVRI & LSHTM Uganda Research Unit Clinical Laboratory using the Cobas e 411 analyzer (Roche Diagnostics, Mannheim, Germany) and the Elecsys HBsAg II, Anti-HBc II, and Anti-HBS II kits. The HBV results were categorized into one of five groups: 1) actively (acute or chronic) infected (HBsAg positive), 2) previously infected or resolved infection (HBsAg negative, anti-HBc & anti-HBs positive), 3) vaccinated (anti-HBs positive, HBsAg & anti-HBc negative) or 4) susceptible or HBV naive (HBsAg, anti-HBc & anti-HBs negative). A fifth group included those with indeterminate results (HBsAg negative, anti-HBs negative, anti-HBc positive) whose categorization can be either of four states namely, resolved infection (most common), false-positive anti-HBc, low-level chronic infection, or resolving acute infection [17]. Samples in this group were retested on all three HBV tests to rule out false-positive anti-HBc results and thereafter categorized as resolved or previously infected if still anti-HBc positive. The anti-HBs titer cut-off value was 10 IU/L.

### Determination of hepatitis B virus prevalence and incidence

To determine HBV incidence, samples were tested at two time points namely at baseline and follow-up visits which were approximately 12 months apart. Initially, all samples from the follow-up visit (*n* = 467) of the parent cohort study were tested. After this testing, samples whose results were consistent with active or resolved infection had their corresponding baseline samples tested. Follow-up samples whose corresponding baseline samples were negative for all HBV assays were subsequently identified as HBV incident cases. Samples that were negative by all HBV assays at the follow-up visit did not have their corresponding baseline sample tested. To better estimate HBV prevalence at baseline, we also tested baseline samples from those study participants who were not seen at the follow-up visit because they were lost to follow up (*n* = 50). Person time was calculated as the amount of time between test visits, with those incident cases estimated as having acquired HBV at the midpoint of their follow up time. We report baseline prevalence as a percent (with binomial standard errors), and incidence as cases per 100 person years with 95% confidence intervals.

### Data analysis

Bivariate analyses were used to assess the potential association between exposure variables (demographics and socio-behavioural characteristics) and HBV infection using chi-square tests and unadjusted logistic regression models. Factors that were significant at bivariate analysis with *p* ≤ 0.2 were added in the multivariable logistic regression model to estimate the adjusted odds ratios. Exposure variables were added to the model one at a time and their effect on model adequacy determined by the log-likelihood tests to compare the fit of the models with and without the variable. Confounding was assessed by the change of magnitude in the effect measure with the addition and removal of new variables. In the final adjusted model, variables with a *p*-value < 0.05 were taken to be associated with the risk of HBV infection. Data analysis was performed using Stata® 14 (StataCorp, College Station, Texas).

## Results

### Study participant characteristics

A total of 517 participant samples were tested for HBV of which 272 (52.6%) were from Kasenyi landing site and 245 (47.4%) from Jaana Island. Of these, 278 (53.8%) were from female participants. The mean and median age of the study participants was 31.1 (SD ± 8.4) and 30 (IQR 25–37) years respectively. About a quarter (24.2%) of the participants were aged 13–24 years and the rest were above 25 years. The majority (52.6%) of the participants were engaged in fishing, fishing-related business, or trade.

### Hepatitis B prevalence

Of the 517 study participants, 246 (47.6%, SE: 2.2%) had evidence of exposure to HBV, including 36 (7.0%, SE: 1.1%) with active (acute or chronic) infection and 210 (40.6%, SE: 2.2%) with resolved or prior infection (Table 1). Active hepatitis B infection prevalence in Jaana Island was about 2.5 times that of Kasenyi mainland (10.2% vs 4.0%). None of the participants engaged in farming, those whose occupation was categorised as housewives, or who reported not having sexual partners within the last 12 months had active HBV infection. Prevalence of active HBV infection was highest in participants working in bars and lodges (10.6%) followed by those in fish or fish related business (7.8%). Active HBV infection among married participants was twice that of unmarried ones.

**Table 1:** Hepatitis B prevalence in Lake Victoria fishing communities

Characteristic	Total	Active HBV infection	Resolved HBV infection	HBV vaccinated	HBV naive
	N	n (%)	n (%)	n (%)	n (%)
	517	36 (7.0)	210 (40.6)	20 (3.9)	251 (48.6)
**Age (years)**					
13–24	125	7 (5.6)	33 (26.4)	6 (4.8)	79 (63.2)
25+	392	29 (7.4)	177 (45.2)	14 (3.6)	172 (43.9)
**Sex**					
Female	278	14 (5.0)	108 (38.8)	14 (5.0)	142 (51.1)
Male	239	22 (9.2)	102 (42.7)	6 (2.5)	109 (45.60
**Occupation**					
Fishing & related	219	17 (7.8)	105 (47.9)	7 (3.2)	90 (41.1)
Trade/Business	53	4 (7.5)	18 (34.0)	1 (1.9)	30 (56.6)
Bar/Lodge	47	5 (10.6)	17 (36.2)	0 (0.0)	25 (53.2)
Farming	23	0 (0.0)	6 (26.1)	4 (17.4)	13 (56.5)
Housewife	46	0 (0.0)	17 (37.0)	3 (6.5)	26 (56.5)
Others	129	10 (7.8)	47 (36.4)	5 (3.9)	67 (51.9)
**Marital status**					
Not Married	204	9 (4.4)	78 (38.2)	7 (3.4)	110 (53.9)
Married monogamous	239	21 (8.8)	102 (42.7)	11 (4.6)	105 (43.9)
Married polygamous	74	6 (8.1)	30 (40.5)	2 (2.7)	36 (48.6)
**Sexual partners in the past 12 months**					
No	79	0 (0.0)	27 (34.2)	3 (3.8)	49 (62.0)
Yes	438	36 (8.2)	183 (42.8)	17 (3.9)	202 (46.1)
**New sexual partner in the past 12 months**					
No	297	29 (9.8)	121 (40.7)	13 (4.4)	134 (45.1)
Yes	141	7 (5.0)	62 (44.0)	4 (2.8)	68 (48.2)
**Condom use in the past 12 months**					
No	287	22 (7.7)	111 (38.7)	16 (5.6)	138 (48.1)
Yes	151	14 (9.3)	72 (47.7)	1 (0.7)	64 (42.4)
**Community**					
Mainland	272	11 (4.0)	95 (34.9)	15 (5.5)	151 (55.5)
Island	245	25 (10.2)	115 (46.9)	5 (2.0)	100 (40.8)
**Syphilis**					
Positive	39	4 (10.3)	18 (46.2)	3 (7.7)	14 (35.9)
Negative	474	32 (6.8)	189 (39.9)	17 (3.6)	236 (49.8)
**HIV**					
Positive	119	11 (9.2)	58 (48.7)	2 (1.7)	48 (40.3)
Negative	394	25 (6.3)	149 (37.8)	18 (4.6)	202 (51.3)

Study participants involved in fishing or fish related business had the highest prior exposure to HBV (47.9%) while those employed in farming had the lowest (26.1%). Altogether, 251 (48.5%) of the study participants had no evidence of active infection with, prior exposure to, or vaccination against HBV. A total of 20 (3.9%) of the study participants had results indicative of HBV vaccination (anti-HBs positive, anti-HBc and HBsAg negative), majority 14 (70%) of whom were female and from Kasenyi mainland 15 (75%). Those engaged in farming also had the highest proportion of participants (17.4%) with evidence of prior HBV vaccination while those employed in bars and lodges had none in this category.

### Hepatitis B incidence

**Fig. 1:**
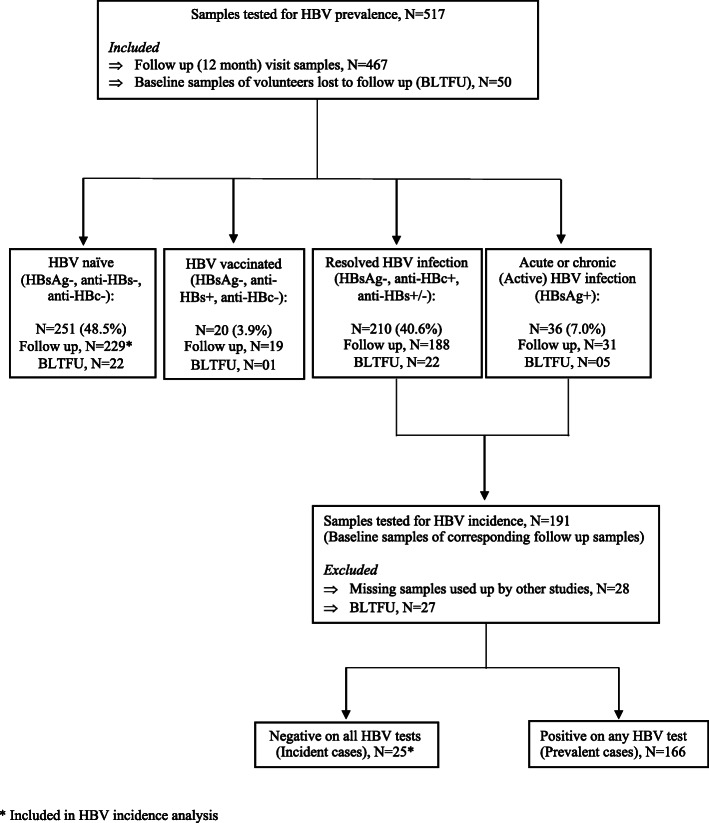
Hepatitis B testing schematic

For the determination of incidence, we tested 191 baseline samples of study participants that were HBsAg or anti-HBc positive at follow-up (Fig. 1). Of these, 25 samples were negative on all baseline HBV tests, representing the incident cases. Together with the 229 samples that were HBV negative at both the baseline and the follow-up visit, we thus observed 25 incident cases over 238-person years to obtain an incidence rate of 10.5 cases/100PY (95% CI: 7.09–15.53).

In bivariate analysis, being above 25 years of age and staying in Jaana Island were significantly associated with HBV incidence and were included in our assessment of predictors of HBV infection. In the multivariable analysis (Table 2) -adjusted for occupation, marital status, sexual partners, and condom use, being above 25 years was associated with a 1.6 times increase in the odds of acquiring HBV (95% CI 1.1–2.2; *p* < 0.01) and staying in Jaana Island was associated with a 1.4 times increase in the same odds (95% CI 1.1–1.8; p < 0.01). Having a sexual partner in the last 12 months or being HIV positive were borderline significant risk factors for the acquisition of HBV in the bivariate analysis but non-significant after multivariable analysis. Occupation, marital status, having a new sexual partner in the last 12 months, condom use in the last 12 months, and having syphilis were not associated with HBV incidence.

**Table 2:** Hepatitis B acquisition risk factors in Lake Victoria fishing communities

Characteristic	Unadjusted Risk Ratios			Adjusted Risk Ratios		
	RR	95%CI	P-value	RR	95%CI	P-value
**Gender**						
Female	1 (Ref)					
Male	1.2	(0.9–1.5)	0.19			
**Age (years)**						
13–24	1 (Ref)					
25+	1.6	(1.2–2.3)	0.004	1.6	(1.1–2.2)	0.01
**Occupation**						
Trade/business	1 (Ref)					
Fishing & related	1.3	(0.9–2.1)	0.20			
Bar/lodge	1.1	(0.6–2.0)	0.62			
Farming	0.6	(0.3–1.6)	0.26			
Housewife	0.9	(0.5–1.7)	0.47			
Others	1.1	(0.7–1.7)	0.65			
**Marital status**						
Not Married	1 (Ref)					
Married monogamous	1.2	(0.9–1.6)	0.18			
Married polygamous	1.2	(0.8–1.7)	0.51			
**Any sexual partners in the past 12 months**						
No	1 (Ref)					
Yes	1.5	(1.0–2.2)	0.06			
**New sexual partner in the past 12 months**						
No	1 (Ref)					
Yes	1.0	(0.8–1.4)	0.79			
**Condom use in the past 12 months**						
Yes	1 (Ref)					
No	0.8	(0.6–1.1)	0.14			
**Community**						
Mainland	1 (Ref)					
Island	1.5	(1.1–1.9)	0.003	1.4	(1.1–1.8)	0.01
**Syphilis testing at baseline**						
Negative	1 (Ref)					
Positive	1.2	(0.8–1.9)	0.39			
**HIV testing at baseline**						
Negative	1 (Ref)					
Positive	1.3	(1.0–1.7)	0.06			

## Discussion

In this study, we investigated the prevalence, incidence, and risk factors of HBV acquisition in selected fishing communities of Lake Victoria, Uganda. Our results show that 7% of the study population in these communities was infected with the HBV and about 41% had evidence of prior infection with this virus. The results further showed that HBV incidence in these communities was very high at over 10.5 cases per 100 person-years and that incident infection is associated with living on an isolated island community.

The 7.0% active HBV infection prevalence observed in our study is slightly lower than that reported by several studies conducted in Uganda before 2016. These studies reported a prevalence of between 8.1% in health care workers from the centrally located Mulago national referral hospital to 14.9% in adults from Northern Uganda [18–21]. It was also lower than the 2005 national HBV prevalence of 10.6% obtained from a hepatitis B serosurvey that was incorporated into the Uganda HIV/AIDS Sero-Behavioural Survey but comparable to the 6.2% reported by the same survey for the Central region where our study population is located [10]. Our study’s prevalence was however substantially higher than what has been reported in more recent studies including the UPHIA 2016–2017 national serosurvey and a 2016 HBV modelling study which estimated a national HBV prevalence of 4.3 and 5.5% respectively [3, 9]. In the area where our study communities are located, the HBV prevalence reported by the UPHIA 2016–2017 national serosurvey was 1.6% suggesting that as of 2015 when samples for our study were collected, the HBV prevalence in the Lake Victoria fishing communities was about four times higher than that of the general population surrounding these communities.

Nearly half (47.6%) of the population was either previously exposed to or actively infected with HBV and 3.9% had serological evidence of HBV vaccination. The remaining 48.5% had never been infected or vaccinated against HBV. These results suggest that about half of the population in the fishing communities of Lake Victoria in Uganda may be susceptible to HBV infection. Given the very high HBV incidence we observed, universal hepatitis B vaccination which was rolled out in 2020 by the Ministry of Health should be prioritised for this community [22, 23].

Our data indicated that HBV prevalence was significantly higher in the island community compared to the mainland community. The national serosurveys of 2005 and 2016/17 also reported large variations in HBV prevalence across the country even though the reasons for this variance were not conclusively explained [9, 10]. However, in the Central region where our study’s two fishing communities are located, the HBV prevalence was reported at 1.6% by the UPHIA 2016–2017 national serosurvey which is substantially lower than what our study found. We compared the hepatitis B infection risk factors across the two study communities using the HBV risk factors that were collected but none could explain the difference in HBV acquisition risk between our study communities.

A very high HBV incidence of 10.5 cases/100PY was observed in our study population. To the best of our knowledge, Hepatitis B virus incidence has never been reported before in this population. Indeed, there is a paucity of data on HBV incidence in the various subpopulations of Uganda or the sub-Saharan region in general. Wahome et al. reported an HBV incidence rate of 6.0/100 person-years (95% confidence interval [CI], 3.9–9.1) in HIV-1 negative men who have sex with men in Kenya [24]. Compared to Wahome’s study group which reported high-risk sexual behaviour, HBV incidence in our study population was nearly twice as high. The reasons for this high HBV incidence are not clear. Most HBV infections occurring within the Ugandan populations have been attributed to sexual contact [10, 25]. Considering that a high HIV incidence that has been reported before in these fishing communities [11, 12], it is possible that most HBV transmissions in our study population also occurred through sexual contact. Surprisingly, there was no statistically significant association between HBV incidence and condom use or sexual partners in our study. This could be due to the underreporting of these variables by our study participants.

Among the different characteristics of this study population that were examined, being above 25 years of age and staying in Janna Island community were significant risk factors for the acquisition of HBV after adjusting for other factors. Similar to what was observed in this population with HIV infection, marital status, having a new sexual partner in the last 12 months, and condom use were not significant risk factors of transmission of HBV [11]. Staying in the island community of Jaana carried a significantly higher risk of acquisition of HBV compared to staying on the mainland. A possible explanation for this difference is that there is easier access to better health facilities on the mainland compared to the island. Access to health facilities could facilitate the treatment of sexually transmitted infections which are a risk factor for the transmission of HBV. Other factors that could explain this difference in HBV infection risk could be differences in sexual partners, sexual practices, or risk behavior. An examination of the available data did not suggest that there were significant differences in these factors between the two study sites. However, the percentage of study participants that had sexual partners in the last 12 months was higher in Jaana (92%) compared to Kasenyi (78%) possibly putting the former at a higher risk of HBV infection compared to the latter.

Similar to what has been shown for HIV in the fishing community, there was no association between HBV incidence and occupation [12, 26]. However, it is worth noting that prevalence was highest among the bar workers which is in concordance with what was reported for HIV. This is likely because bar workers in the Lake Victoria fisherfolk community usually also engage in commercial sex work, a high-risk factor for the acquisition of HBV [26].

This study had some limitations. Only two study communities were selected as representative communities of the Lake Victoria fishing communities. Whereas both an island and a mainland community with the best retention were chosen from a 10-community study (to allow for more power to calculate incidence), they may not have been an accurate representation of the different Lake Victoria fishing communities. Secondly, the study population did not include young children below 13 years who might have a lower HBV prevalence due to the introduction of routine infant hepatitis B virus vaccination in the Uganda national immunisation program [10]. Given that our study participants were above 13 years, born before this vaccination program was instituted, our results do not reflect the impact of this intervention on HBV prevalence and incidence in these communities. We also recognize that our sample size was modest, and with 254 volunteers and 25 incident cases of HBV identified, we have limited power to see correlates of incidence, were they to exist. Lastly, some risk factors for the acquisition of HBV such as a history of blood transfusion, tattooing, intravenous drug use, and sexually transmitted infections apart from syphilis were not examined because they were not collected in the parent cohort study. Among our study population, however, sexually transmitted infections would be of most relevance and possibly of significant impact on the study outcomes considering that blood transfusion, tattooing, and injection drug use is uncommon in these communities.

## Conclusion

In conclusion, the HBV incidence in Lake Victoria fishing communities of Uganda is very high, particularly in the island communities. Factors contributing to a higher HBV incidence in the Island communities compared to the mainland need to be investigated further. The majority of the population in the fishing communities of Lake Victoria is susceptible to HBV infection and would therefore benefit from prevention information and vaccination against this infection.

## Data Availability

The datasets used and/or analysed during the current study are available from the corresponding author on reasonable request. A full data set containing the data supporting the study findings in this article can also be obtained from the Program Data Manager, by email to tnakaweesa@iavi.or. ug or information@iavi.or.ug.

## Abbreviations

anti-HBs: Hepatitis B surface antibodies
HBsAg: Hepatitis B surface antigen
HBV: Hepatitis B virus
anti-HBc: Total hepatitis B core antibody
UNCST: Uganda National Council for Science and Technology
UPHIA: Uganda Population-based HIV Impact Assessment
UVRI-REC: Uganda Virus Research Institute Research Ethics Committee
